# Measles Epidemiology and Outbreak Investigation Using IgM Test in Laos

**DOI:** 10.2188/jea.11.255

**Published:** 2007-11-30

**Authors:** Chushi Kuroiwa, Phengta Vongphrachanh, Phoxay Xayyavong, Kongmany Southalack, Masahiro Hashizume, Satoshi Nakamura

**Affiliations:** 1 Bureau of International Cooperation, International Medical Center of Japan, and Japan International Cooperation Agency in Lao PDR, and Department of Epidemiology, Juntendo University.; 2 National Center for Laboratory and Epidemiology, Ministry of Health, Laos.; 3 Local staff for Japan International Cooperation Agency in Laos.; 4 Department of International Community Health, Graduate School of Medicine, the Tokyo University.; 5 Research Institute, International Medical Center of Japan.

**Keywords:** measles elimination, Laos, routine immunization

## Abstract

Following the Pan American Health Organization (PAHO) recommendation on measles elimination, the Western Pacific Region of WHO (WPR) is emphasizing accelerated measles control programme especially since the achievement of polio eradication in WPR in 2000. This includes upgraded surveillance and mass measles vaccination campaign for children aged 9 months to 4 years. However, there are limited scientific evidences supporting the feasibility of this programme in Laos. To examine measles elimination feasibility in the country, we conducted measles outbreak investigation using immunoglobulin M (IgM). From March 1999 to March 2000, we conducted 7 outbreak investigations. At the outbreak sites, we examined clinical manifestations of cases and collected individual data. About five blood samples were drawn from each outbreak, and IgM antibodies to measles were tested. Of 7 investigated outbreaks, 5 were confirmed as measles, one was chickenpox, and one occurred in the inaccessible area due to flooding. In a village of high land Lao, blood drawn was refused. Of 185 cases, 64 (34.6%) cases were immunized, and 110 (59.5%) were unimunized. The estimated vaccine efficacy is 67.9%. The number of measles cases among school-aged children was 74 (40.0%), which represented 2.5% of the total population in investigated villages. Our findings showed various difficulties of the surveillance and the limited outcomes of mass measles vaccination campaign under the accelerated measles control programme by WPR. Efforts to improve cold chain as well as increasing routine immunization coverage must be the priority of measles control.

After the achievement of regional polio eradication in the Pan American Health Organization (PAHO) in 1994^1^^)^, the progress towards measles elimination from Americas was reported^2^^, ^^3^^)^. The strategies include: 1) implementation of national catch-up vaccination campaign targeting children 9 months to 14 years of age regardless of history of measles disease or vaccination, 2) ensuring routine immunization coverage to every new cohort of children to at least 90% with one dose of measles vaccine, 3) implementation of periodic follow-up vaccination campaign in order to avoid accumulation of susceptible persons over time, 4) the establishment of case-based surveillance with laboratory confirmation to timely detect measles circulation.

The Western Pacific Region of WHO (WPR) also achieved regional polio eradication on 29 October 2000^4^^, ^^5^^)^. And efforts for measles elimination as a form of accelerated measles control programme^6^^)^, which has been conducted in WPR since around 1996^7^^, ^^8^^)^, began to be strengthened. There are three stages in measles surveillance according to the guideline^9^^, ^^10^^)^ : 1) Control phase: When measles is endemic, routine monthly reporting of aggregated data of clinical measles cases from peripheral to intermediate and central level is recommended. Only outbreak (not each case) should be investigated. 2) Outbreak prevention phase: When low incidence of measles is achieved with periodic outbreaks due to accumulation of the susceptible, routine monthly reporting of aggregated data of clinical measles cases is recommended from peripheral to intermediate and central level. All suspected outbreaks should be investigated immediately and case-based collected. Suspected measles epidemics should be confirmed by conducting serology on the few cases only. 3) Elimination phase: Case-based surveillance should be conducted and every case reported and investigated immediately from peripheral level to intermediate level including weekly reporting system. Laboratory specimens should be collected in every case.

Based on the experience in the Americas, Quadros et al recommended initiation of measles elimination, which is implementation of national catch-up vaccination campaign, should be after the achievement of the reduction of measles cases and high routine immunization coverage of 90%^2^^)^. However, Laos, in which there were many measles cases and where measles immunization coverage has been very low, started its preparation for measles elimination programme in the form of a pilot mass vaccination campaign in 2 provinces of 18 provinces in March, 2000^11^^)^, and it planned to expand to all provinces in 2001; besides, the target population is children aged 9 months to 4 years^7^^)^, which is similar to “vaccination of children 6 months to 5 years of age” in Cuba, which allowed large epidemics^2^^, ^^12^^)^.

Laos is in the control phase of measles elimination programme. There are only numbers of clinical measles cases by province from 1994. Thus data on the number of confirmed measles cases, vaccine history, age distribution and vaccine efficacy are not available. We conducted measles outbreak investigation using immunoglobulin M (IgM) to examine the feasibility of measles elimination in the country or to clarify the feasibility of transition from control phase to outbreak prevention phase.

## METHODS

### Geography, and ethnic groups

Laos is a land-locked country with an estimated population of 5 million^13^^)^. Because of geographic difficulties with a lot of mountains and bad road conditions, about half of the roads at the district level are not accessible by car or motorcycle during the rainy season from April to October^14^^)^. There are 47 identified ethnic groups, and “Lao Loum” comprises 50.5% of the population, and “Hmong”, the majority of high land Lao (Hmong, Kaw, Yao, and Phu Noi), comprises 6.47%; besides, ethno-linguistic nomenclature in the literature about the Laos varies widely and creates confusion and there are officially five major language groups^15^^)^, which sometimes become a hurdle for ethnic groups understanding health education.

### Surveillance

In 1992, monthly report of measles from provincial level to central level or National Center for Laboratory and Epidemiology (NCLE) was initiated as part of acute flaccid paralysis (AFP) surveillance for polio eradication^16^^, ^^17^^)^, and weekly report from all provinces was established in 1994. The data of weekly report include the number of measles cases and deaths in each province, and individual information on the age, location, and immunization status are not available or incomplete, unless case investigations are conducted.

### Expanded Programme on Immunization (EPI)

The EPI was initiated in 1979. By 1982, EPI was operating in only 2 provinces and 10 districts. By 1992, it expanded to 97 (80%) of the 121 established districts at that time^18^^)^, and covered all districts in 1993. Immunization coverage with measles increases from 29% in 1990 to 73% in 1996, and declined to 65% in 1999. Routine measles vaccination is provided children aged 9 to 23 months.

### Survey design

We analyzed weekly report from January 1994 to October 2000 with number of cases and deaths sending from provinces to NCLE.

From March 1999 to March 2000, 18 big outbreaks (more than 20 cases) were reported from provinces by weekly report, and among them we conducted 7 outbreak investigations, in which we visited 5 provinces, 7 districts and 9 villages (table1). Of 18 outbreaks, 7 were inaccessible (table 2). The team was consisted of a national surveillance staff, two laboratory staff from NCLE, and two experts (a Japanese and a Laotian pediatrician) from central level, and in the outbreak site, a provincial staff (EPI or surveillance manager) and a district health staff were joined the team. At the provincial and the district hygiene station, individual data was incomplete or not available. At all investigated sites, teams went from door to door. The pediatricians examined clinical manifestations of all cases and the surveillance staff collected individual data on the age, immunization status and past history of measles. At each investigated site, after parents of case or cases provided written informed consent, the laboratory staff chose randomly about five cases who had onsets within one month, and drew blood samples from them. The centrifuged samples were kept in the laboratory at each provincial hospital until the team left the province for Vientiane. IgM antibodies to measles were tested at NCLE in Vientiane using an enzyme immunoassay kit for qualitative determination of IgM antibodies to measles virus (MEASLES IgM (II) - EIA “SEIKEN”). The data of final classification were sent back to the provincial hygiene stations as feedback.

**Table 1.** Measles outbreak investigation using IgM test from March 1999 to March 2000.

Number	Report Week (month) / year	Number of cases/death in weekly report	Village	District	Province	Date of investigation	Number of investigated coses	Clinical diagnosis	Antibody Positive/total	Final diagnosis	Remarks
1	7(Feb)-10(March) / 1999	23 / 0	phonsoung	Khanthaboury	Savanakhet	11-Mar-99	27	Measles	4/5	Measles	
2	3(Jan)-8(Feb) / 1999	175 / 4	Boualapha	Boualapha	Khammuane	12-Mar-99	11	Measles	2/2	Measles	
			Napeng	Boualapha	Khammuane	12-Mar-99	47	Measles	5/5	Measles	
			Namdik	Hinboun	Khammuane	13-Mar-99	35	Measles	5/5	Measles	
3	12(March) / 1999	76 / 11	Song	Kham	Xiengkuang	21-Apr-99	1(20)*	Measles	NA	Measles	can not draw blood
4	16(Apr) / 1999	31 / 0	Tho	Phalanxay	Savanakhet	18-May-99	45	Measles	11/11	Measles	
5	31(Jul) / 1999	87 / 4	Kongnanyai	Sanxay	Attapeu	Jul-99	NA			NA	can not reach the district due to flood
											
6	44(Oct) / 1999	48 / 0	Houayhang	Thapangthong	Savanakhet	12-Nov-99	7	Measles	5/5	Measles	
			Napasat	Thapangthong	Savanakhet		13	Measles	7/7	Measles	
7	7(Feb) / 2000	22 / 0	Konlang	Pakbeng	Oudomsay	2-Mar-00	30	Chicken pox	0/5	Chiken pox	

* Of 20 cases, only one case was interviewed

NA: Not available

**Table 2.** Inaccesible measles outbreak investigation during the survey period.

Number	Report Week (month) / year	Number of cases/death in weekly report	District	Province	Reasons
1	17(April) /1999	154/12	Boualapha	Khammuane	Road suspension due to flood
2	19(May) /1999	130/2	Hongsa	Sayyabuly	Very bad road in moutain
3	24(June) /1999	148/2	NA	Phongsaly	2 days walk to the village
4	31(Jul) /1999	87/4	Sanxay	Attapeu	Road suspension due to flood
5	39(Sep) /1999	48/0	Sayyabuly	Sayyabuly	6 hours walk to the village
6	43(Oct) /1999	94/2	NA	Sayyabuly	2 and a half-days walk to the village
7	46(Nov) /1999	53/0	Sanxay	Attapeu	Road suspension due to flood

NA: Not available

## RESULTS

**Figure 1.**
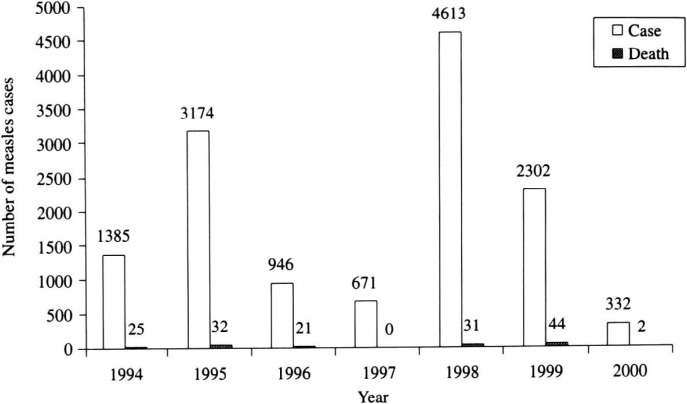
Reported measles cases in Laos from 1994 to 2000.

**Figure 2.**
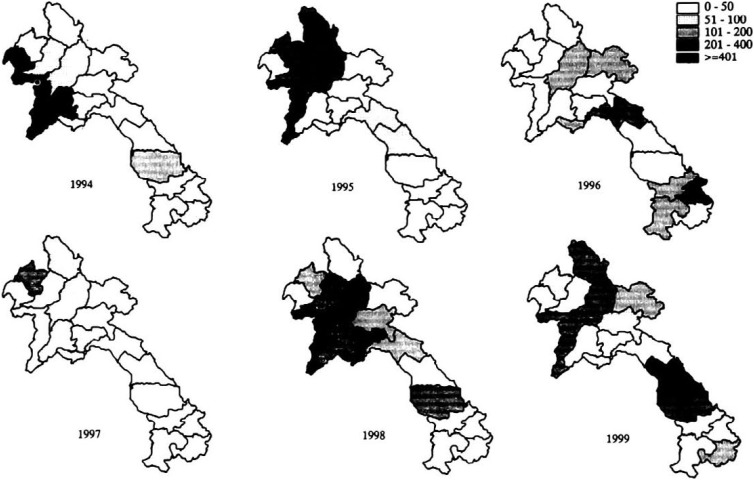
Measles cases by province, Laos from 1994 to 1999, by weekly report.

We clarified measles epidemic in the country. As shown in figure 1, measles cases increased every three years peaking at 3174 cases in 1995, and 4613 cases in 1998, and the case fatality rate was 1% (32) in 1995, and 0.7% (31) in 1998. Figure 2 shows measles cases by province from 1994 to 1999. The outbreaks in northern part in 1994 and 1995 were observed, and after 2 years of low incidence, another outbreaks in the North in 1998 and in the South in 1999 are shown.

**Figure 3.**
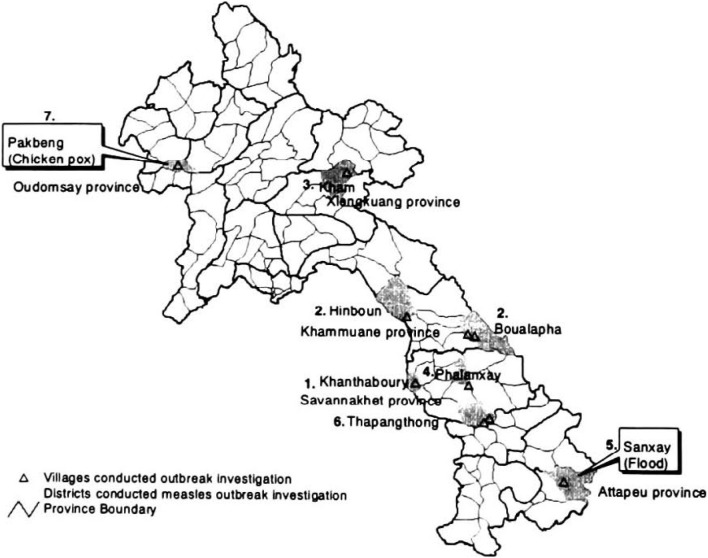
Districts conducted measles outbreak investigation, from March 1999 to March 2000. Numbers 1 to 7 are the outbreak investigation numbers shown in table 1.

As shown in table 1 and figure 3, among 18 big outbreaks with more than 20 reported measles cases, we responded to 7 reported outbreaks, and did not respond to 7 (39%) outbreaks (table 2) because of road suspension, long-walk distance, and very bad road in mountainous area, indicating serious difficulty of access in the country. Of 7 outbreaks, 5 were confirmed as measles, one was misdiagnosed as chickenpox, and one was not accessible due to flood-induced road suspension. Of 5 confirmed measles outbreaks, 4 outbreaks in 7 villages were confirmed by IgM antibody and clinical diagnosis, and one outbreak was confirmed only by clinical diagnosis.

Only one outbreak was clinically misdiagnosed. The outbreak in Konlang village in Pakbeng district of Oudomsay province (No.7) was initially investigated by health workers at the dispensary and diagnosed as measles, but we found clinical features of the cases on the skin showing the typical manifestations of chickenpox; from macule to papule to vesicle to scabs, and all IgM antibodies of five samples were negative. Although we left for Kongnanyai village in Sanxay district of Attapeu province (No. 5), we had to give up accessing the outbreak site, because of the suspension of the road due to flood caused by heavy rain in rainy season, which information was not received until we arrived in the province indicating poor communication in the country.

The difficulty in IgM test and strong infectivity of measles were revealed in a minority village. The clinically confirmed outbreak occurred in Song village of high land Lao of Hmong people in Kham district of Xiengkuang province (No. 3), and we walked one and a half hour to reach the village in a mountainous area. In the village, we found 20 clinical measles cases, and we asked a case for blood drawn but parents refused it because they were afraid of their child’s death due to blood drawn. After this refusal, the other parents surrounding us took back their children to their houses, so we interviewed only one case. The official report shows extremely high fatality rate of 14.5% (11/76) in this outbreak, which occurred in this village and surrounding 3 Hmong villages, and about one-hour walk brings village people to next village.

**Figure 4.**
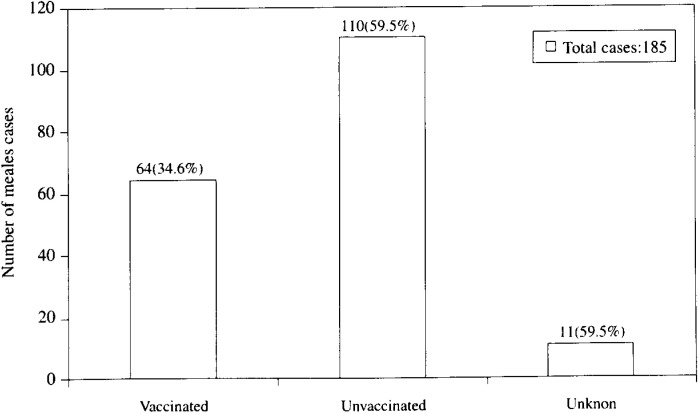
Measles immunization history by measles outbreak investigation, from March 1999 to March 2000.

Figure 4 shows the proportion of immunization status of the 185 measles cases confirmed by IgM antibody and clinical diagnosis by the pediatricians. This is total of number of investigated cases in number 1, 2, 4, and 6 in table 1. Of 185 cases, 64 (34.6 %) received measles immunization before the outbreaks occurred, 110 (59.5 %) cases had no immunization history, and 11 (5.9%) were unknown. Based on the limited data that we obtained by this survey and national immunization coverage, we calculate the vaccine efficacy^19^^)^ of the children aged 1 to 4 years in the country. A total population of the investigated villages is 2871, and the estimated number of the children aged 1 to 4 years is 374, which is 13% of the total population^20^^)^. Because it was difficult to have measles immunization coverage for each year for each village, we used national measles immunization coverage for children 12 to 23 months old: 73%, 67%, 67%, and 65% for 1996, 1997, 1998, and 1999 respectively. The average is 68%, which roughly represents measles immunization coverage for children aged 1 to 4 years. Of 185 investigated cases, there were 92 measles cases aged 1 to 4 years, of which 34 (36.9%) were immunized, 50 (54.3%) were unimmunized, and 8 were unknown. Thus, attack rate in immunized children; 34 / (374-8) × 0.68 = 0.137. Attack rate in unimmunized children: 50 / (366 - 366 × 0.68) = 0.427. Therefore, estimated vaccine efficacy: (0.427 - 0.137) /0.427 × 100 = 67.9%. This figure indicates the possible failures in the cold chain.

Laos is a country with a population of 5 million and 200,000 births (4% of the total population) per year^20^^)^. The estimated vaccine efficacy is 67.9%, and the average measles coverage in the past four years is 68%. This implies that only 92,000 (200,000X0.679X0.68) children (46%) of the newborn will be protected against measles and 108,000 children (54%) will remain susceptible. Thus, each year an additional 108,000 children will be the pool of susceptible persons, and in less than two years, the cumulative number of susceptible children will reach the number of children in one birth cohort.

**Figure 5.**
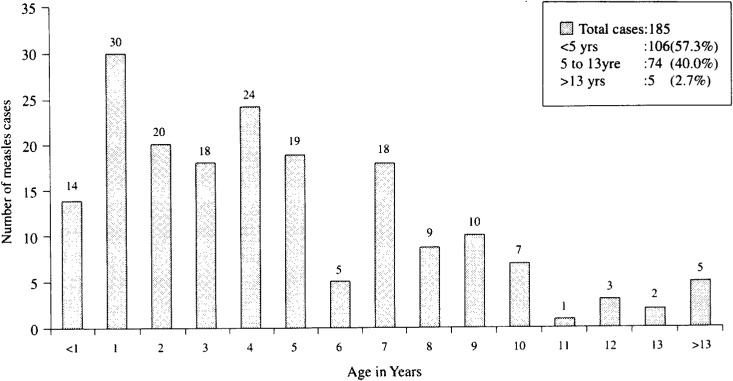
Age distribution of measles cases by measles outbreak investigation, from March 1999 to March 2000.

Figure 5 shows age distribution of the measles cases. Of 185 cases, 74 (40.0 %) were school-aged children, and 106 (57.3 %) were under 5 years old. Cases of school-aged children was 2.5% (74/2871) of the total population in investigated villages. There were 5 cases of children over 13 years.

We estimate the number of reduction of measles cases by the mass measles campaign. Mass polio vaccination campaign with oral poliovirus vaccine (OPV) achieved coverage of around 90% nationwide^21^^)^. Since measles vaccination is administered by injection, we estimate measles vaccine coverage to be a little lower at about 85%. Routine immunization requires four vaccines and measles campaign requires only one, so estimated vaccine efficacy by the mass campaign would increase coverage to around 80% (12.1% more than by routine immunization). By measles campaign, we expect 68% (0.85X0.8) of the target population would be protected against measles. With routine immunization coverage at 68% and vaccine efficacy at 67.9%, we get a 46% (0.68X0.675) measles coverage of children aged 1 to 4 years. Based on the experience from the polio eradication activities, most of all children who had received vaccines by routine immunization also received vaccines by mass campaign, so 22% (68-46) of the target population would increase immunity to measles by the mass measles campaign. Our data showed immunized children aged 1 to 4 years of the total measles cases accounting for 36.9%, so about 8% (0.369X0.22) of the total measles cases would be reduced. 2302 measles cases occurred in 1999, so if mass measles vaccination campaign for children aged 9 months to 4 years had been conducted, only 184 cases (2302X0.08) would have reduced.

## DISCUSSIONS

Measles clinical diagnoses were confirmed by IgM antibody in 7 villages of the total 9 investigated villages (78%), which suggests relatively high reliability of measles diagnosis by health workers and/or doctors at the community level, and the necessity of IgM test for more reliable diagnosis if the country proceeds to outbreak prevention phase. All outbreak investigation also enables to obtain case-based data on the age, location, and immunization status which are not available or incomplete at provincial and district level.

However, our findings also showed various difficulties in implementation of outbreak prevention phase, in which measles incidence decline to low level and outbreak investigation and serological confirmation in each outbreak are required. Measles is still endemic in Laos, and outbreak movements of measles by year are clearly seen in the map. Inaccessibility to outbreak sites due to geographical difficulty makes impossible to implement all outbreak investigation, and the refusal of the blood drawn in Song village of high land Lao of Hmong people suggests the extreme difficulty in serological confirmation for each outbreak. High fatality (14.5%) in the village and surrounding 3 villages also suggests that extremely low immunization coverage, indicating the strong fear of the injection. However, accelerated measles control programme requires 3 injections of measles vaccine. Routine immunization, mass vaccination campaign and follow-up vaccination campaign, would have negative impact especially for high land Lao people. Besides, this outbreak in these Hmong villages between which people have to walk one hour revealed strong infectivity of measles, which had never been observed during polio eradication activities in the country before the regional declaration of polio free. This suggests the tremendous difficulty in cutting measles virus transmission in high-risk areas compared to poliovirus.

Quadros et al wrote if the initial national mass vaccination campaign was not followed by ongoing routine immunization and follow-up vaccination campaign for children aged <5 years, the number of susceptible children would increase rapidly and measles epidemics will return^2^^)^. Our results showed the cumulative number of susceptible children would reach the number of children in one birth cohort in less than two years in Laos. Besides, our survey revealed the number of measles children aged 5 to 13 years was 2.5% of the total population of investigated villages, which would remain the susceptible population. Thus, even after implementation of the national mass vaccination campaign^7^^)^, measles outbreak in Laos would occur next year (the susceptible: 4%X0.54+2.5% = 4.66% >4%), and will be repeated every less than 2 years due to rapid accumulation of susceptible pre-school-aged children.

Although PAHO recommended the target population of mass measles campaign is children aged 9 months to 14 years^2^^, ^^3^^)^, WPR’s target is children aged 9 months to 4 years and the proposed plan in WPR explains this mass campaign can reduce measles morbidity and mortality^7^^)^. However, the campaign is difficult to expect remarkable reduction of mortality, because as we showed, even after the campaign, about 54 % of newborn baby will remain susceptible next year, and those children are most valuable to measles infection. Our results showed the difficulty in reducing measles cases effectively by this campaign; only 184 cases (8%) reduction of 2302 cases in 1999 outbreak. This mass campaign requires a series of efforts starting from the preparation by central staff and local staff to implementation of mass vaccination by nationwide vaccinators to monitoring by central staff, and these efforts have to be conducted intensively in dry season of 6 months period together with routine immunization services. Besides, this campaign requires tremendous amount of cost: measles vaccines which requires 4 times of routine immunization, disposable syringes for all target children, and operational cost for all nationwide vaccinators. Most of the fund will be supported by overseas donors but it is also impossible for the government of Laos to continue to depend on the supports forever. Thus, this reduction rate of 8% should be judged as a very small figure unless measles elimination is confirmed to be feasible. We must reconsider how much important the routine immunization is.

The proposed plan explained that the same approaches of polio eradication would provide a period of low transmission during which routine coverage can be improved^7^^)^. However, our experiences of polio eradication in Laos^5^^, ^^17^^)^ revealed that consecutive mass vaccination campaigns with oral poliovirus vaccine (OPV) were not able to increase routine OPV coverage^5^^)^, so the mass measles campaign also has a risk of collapsing measles routine immunization. The workload for local staff has become tremendously heavy due to dual eradication programme, and measles control programme should not be impediment to the achievement of global polio eradication^22^^)^.

Following the PAHO recommendation on measles elimination, the accelerated measles control programme in WPR began to be strengthened after the declaration of regional polio eradication. However, our data showed the limited outcomes of mass vaccination campaign under low measles immunization coverage and low vaccine efficacy. Various difficulties in proceeding this programme from control phase to outbreak prevention phase were also revealed. Measles elimination seems to be a dream in Laos, and we have to focus on increasing routine immunization coverage and improving cold chain and logistics that must be the core of EPI.
